# Primary hepatic neuroendocrine tumors: comparing CT and MRI features with pathology

**DOI:** 10.1186/s40644-015-0046-0

**Published:** 2015-08-15

**Authors:** Li-Xia Wang, Kan Liu, Guang-Wu Lin, Tao Jiang

**Affiliations:** Department of Radiology, Beijing Chaoyang Hospital, Capital University of Medical Sciences, Beijing, 100020 China; Department of Radiology, Cancer Hospital, Peking Union Medical College, Chinese Academy of Medical Sciences, Beijing, 100020 China; Department of Radiology, Huadong Hospital, Fudan Universityi, ShangHai, 200040 China

**Keywords:** Liver neoplasms, Neuroendocrine tumor, Computed tomography, Magnetic resonance imaging

## Abstract

**Background:**

Primary hepatic neuroendocrine tumors (PHNET) are extremely rare and difficult to distinguish from primary and metastatic liver cancers since PHNETs blood supply comes from the liver artery. This study aims to investigate CT and MR imaging findings of primary hepatic neuroendocrine tumor (PHNET) and correlation with the 2010 WHO pathological classification.

**Methods:**

We examined CT and MRI scans from 29 patients who were diagnosed with PHNET and correlated the data with the 2010 WHO classification of neuroendocrine tumors.

**Results:**

According to the 2010 WHO classification system, PHNETs are divided into three grades based on histological criteria. Grade 1 tumors are singular, solid nodules with enhancement at the arterial phase on CT and MRI scans. In grade 1 tumors, the dynamic-contrast enhancement curve shows rapid wash-in in the arterial phase. Grade 2 tumors can have a singular or multiple distribution pattern, necrosis, and nodule or marginal ring-like enhancements. Grade 3 tumors have multiple lesions, internal necrosis, and evidence of hemorrhage. Portal venous tumor thrombus was seen in one case. As tumor grades increase, the capsule begins to lose integrity and tumor apparent diffusion coefficient (ADC) values decrease(grade 1: 1.39 ± 0.20× 10^−3^ mm^2^/s versus grade 2: 1.26 ± 0.23× 10^−3^ mm^2^/s versus grade 3: 1.14 ± 0.17× 10^−3^ mm^2^/s).

**Conclusion:**

CT and MRI can reflect tumor grade and pathological features of PHNETs, which are helpful in accurately diagnosing PHNETs.

## Background

Neuroendocrine carcinoma (NEC) is rare and originates in the gastrointestinal tract, neuroendocrine cells, and pancreas. Although it resembles adenocarcinoma, well-differentiated NEC behaves biologically in a more benign fashion [1]. However, one report has shown that undifferentiated NECs could possess invasive and metastatic characteristics [2]. Primary hepatic neuroendocrine tumors (PHNET) are rarer than NEC, grow slower, and have the ability to become malignant. Only a minority of patients have carcinoid syndrome. On CT and MRI scans, PHNETs resemble hepatocellular-carcinoma (HCC), but patients with PHNETs have a better prognosis. Despite tumor recurrence, patients with carcinoid syndrome have a satisfactory 5-year survival rate of 74–78 % and a 5-year recurrence rate of 18 % after a hepatectomy [2–5]. Since CT and MRI scans are sensitive in finding primary liver lesions and metastases, these techniques could help clinicians identify suitable therapeutic approaches and further improve the survival rate of patients.

However, there is some controversy surrounding whether CT and MRI scans can predict tumor grade and malignancy. One research group reported that the CT features of hepatic neuroendocrine tumors vary and do not correlate with their pathologic diagnoses [6]. Another report found that well-differentiated primary neuroendocrine tumors resemble echinococcus cysts on CT and MRI scans [7].

In this study, we retrospectively investigated 29 patients diagnosed with PHNET by surgery or puncture and summarized the characteristics of CT and MRI scans as a way to improve the accurate of diagnosis of PHNET. Additionally, we correlated our findings with the 2010 WHO classification of gastrointestinal neuroendocrine tumors.

## Materials and methods

### Study population

Between May 2004 and December 2013, 29 patients (13 males, 16 females; median age: 47.2 years of age) with PHNETs were admitted to Beijing Chaoyang Hospital of Capital University of Medical Sciences. Of these 29 patients, 5 cases had carcinoid syndrome, 7 patients experienced pain in the right upper abdomen, 9 patients complained of abdominal discomfort, PHNETs were identified in 6 patients by routine ultrasound, and 2 patients had liver tumors detected by a chest CT exam. All patients underwent a hepatectomy or an ultrasound- or CT-guided liver puncture. PHNETs were confirmed by pathology and immunohistochemistry. Serum levels of alpha-feto protein(AFP) were normal (less than 40 ng/ml), viral hepatitis B antigen was negative, and evidence of liver cirrhosis was not observed.

All patients underwent CT and MRI scanning and provided written informed consent. The Hospital institutional review board approved this study.

### CT protocol for PHNETs

Abdominal helical CT examinations were performed with multi-detector row scanners (Light Speed Ultra, GE Healthcare, Milwaukee, Wis). Food and water were deprived 4–6 h before enhanced CT examinations. To show contrast between the liver and digestive tract, an oral positive contrast agent (500 ml water combined with 10 ml iopromide, Bayer Healthcare) was used. The patients were in the supine position, scanning range was from the right diaphragm to the kidney, slice thickness was 5 mm with helical scan and the slice thickness of the reconstruction image was 1.25 mm with a pitch of 0.984:1. All patients received nonionic intravenous contrast material (Ultravist, 300mgI/ml, Bayer Technology & Engineering Company Limited, Berlin, Germany), which was administered at a rate of 4 mL/s (90–120 mL total dose) using a mechanical power injector (Medrad, Pittsburgh, PA, USA) set at 230 mAs and 120 kVp. Thereafter, hepatic arterial, portal venous phase, and delayed phase images were acquired with delays of 25 s, 50–60 s, and 2–3 min, respectively. The slice thickness and reconstruction slice thickness were the same with unenhanced CT. Multi-plane reconstruction (MPR) images were obtained at ADW 4.2 workstation (GE Healthcare, Milwaukee, Wis).

### MRI protocol for PHNETs

Eighty-three percent of patients (24 out of 29) underwent abdominal MRI examinations in a 3.0-T system with a phased-array torso coil for signal reception (Magnetom Trio, Siemens Medical Systems, Erlangen, Germany). A respiratory-triggered T2-weighted fast spin-echo pulse sequence with fat-suppressed technique was performed. The acquisition parameters were as follows: TR/TE (msec), 4000–5000/81; flip angle, 140°; field of view, 38 cm; matrix, 256 × 256; number of sections, 30; section thickness, 6 mm; interval; 1 mm; and one signal acquired. A breath-hold T1-weighted fast low angle shot (FLASH) GRE sequence was performed in all patients with a chemical shift technique. The parameters were set as TR/TE (msec), 210/2.45, 210/3.83; flip angle, 65°; field of view, 38 cm; matrix, 256 × 256; number of sections, 30; section thickness, 6 mm; interval, 1 mm; and one signal acquired. Three-Dimensional Volume Interpolated Breath-hold Examination (3D-VIBE) was performed during hepatic arterial, portal venous and delayed phases at 25 s, 60–70 s and 2 min after administration of a gadolinium-based contrast medium (0.1 mmol/kg) at a rate of 3–5 mL/s, followed by a saline flush (20 mL). The parameters were as follows: TR/TE (msec), 3.68/1.22; Flip angle, 10°; field of view, 35 cm; matrix, 256 × 256; number of sections, 80; and acquisition time, 21 s.

Diffusion-weighted MR imaging was performed before dynamic imaging, using a single-shot spin-echo echo planar imaging (EPI) sequence with b factors of 0 and 800 s/mm^2^ along the three orthogonal directions. The sequence was obtained within two breath-holding periods for the whole liver. The parameters included: TR = 4000 ms, TE = 66 ms, matrix = 138 × 128, FOV = 32 to 38 cm adapted to individual body size, twenty-four slices for each b factor for entire liver, thickness = 6 mm, interslice gap = 0.6 mm, average = 2, bandwidth = 1735 Hz/pixel, and acquisition time = 24 s for each breath-holding period. The parallel imaging algorithms (GRAPPA) with an acceleration factor of 2 were added. Spectral fat saturation was employed systematically to suppress the chemical-shift artifacts. ADC maps regarding isotropic images were automatically acquired and all mean ADCs of the lesions were measured on those maps. For patients who did not hold their breath within 24 s, we induced slices for each b factor and added the thickness of the slice and the gap. The acquisition time in scanning the whole liver for hold-breath one time could be reduced to 16 s.

### Data analyses

Two radiologists blindly analyzed dynamic-contrast enhancement curves (arterial and portal phases) and liver lesions on unenhanced and post-enhanced CT and MRI scans. Measurements of attenuation avoided cystic, necrotic and hemorrhagic areas. If tumors were cystic or predominantly cystic, regions of interest (ROI) on unenhanced and post-enhanced CT and MRI scans were placed in the solid area or margins. Regions of interest (ROI) on diffusion-weighted images were carefully identified in the margins or solid area and ADC values were recorded. Experienced pathologists evaluated hematoxylin-eosin (HE) slides and PHNET grades were defined according to the 2010 WHO classification of gastrointestinal neuroendocrine tumors.

Grade 1(G1): the mitotic rate was < 2 per 10 high power field and the Ki-67 index was ≤ 2 %. Grade 2(G2): the mitotic rate was 2–20 per 10 high power field, and the Ki-67 index was 3–20 %. Grade 3 (G3): the mitotic rate was >20 per 10 high power field and the Ki-67 index > 20 %

## Results

Using pathology and the 2010 WHO classification of PHNET, eight cases were defined as G1, 10 cases were defined G2, and 11 cases were defined G3.

All 19 singular lesions (G1 8 cases, G2 7 cases and G3 4 cases) underwent surgical resection. The maximum diameter of the mass was 8 cm with a clear border. Twelve lesions were predominantly solid, one case showed calcification, and the remaining 6 cases were cystic-solid or predominantly cystic.

HE staining of G1 lesions revealed well-differentiated round or ovoid cells arranged in nests and glandular duct or chrysanthemum formation. The mitosis rate ranged from 0 to 2 per 10 high power fields. In G2 lesions, the cell arrangements were irregular, with mild differentiation and minimal nuclei atypia. The mean mitosis rate was 10 (range: 4 to 16) per 10 high power field. G3 lesions were poorly differentiated with large nuclei and the mean mitosis rate was 27 (range: 22 to 40) per 10 high power field.

Histology showed positive staining for the neuroendocrine marker, synaptophysin (Syn) and CT imaging revealed singular lesions (8 cm) with clear borders and irregular margins. We found multiple liver lesions of different sizes. One PHNET case progressed to tumor thrombus in the portal vein with no evidence of liver cirrhosis in the surrounding normal tissue.

**Fig. 1:**
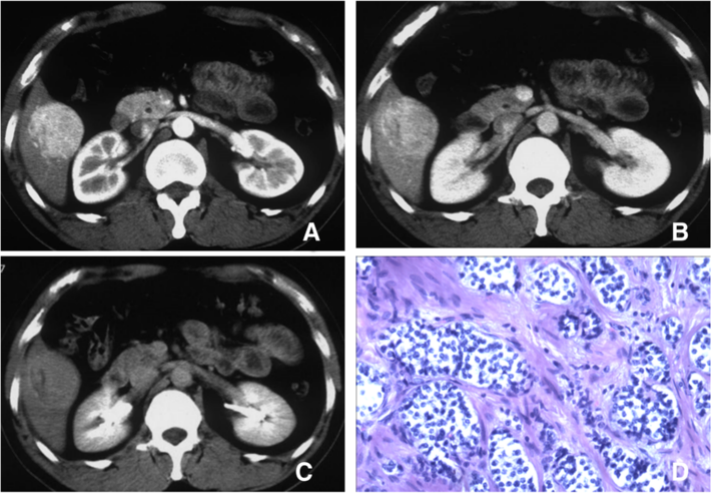
CT scan and histology of grade 1 PHNET. Post-contrast CT image of the arterial phase (**a**), the portal venous phase (**b**) and the delayed phase (**c**) shows marked enhancement in the PHNET relative to the liver parenchyma (right lobe at the arterial phase), and slight hyperattenuation relative to the surrounding liver parenchyma in the delayed phases. The integrity of the capsule is maintained. The mitosis rate shows up as hypo-density and no enhancement on the dynamic enhanced CT scan. HE staining of the tumor (**d**) shows tumor cells arranged as solid nests, consistent cell size and mitosis rate was 1/10 HPF. Magnification: D = 100X

Based on CT imaging (Table 1) and MRI data (Table 2), all G1 liver lesions were singular with only one lesion located in left lobe and remaining lesions the in right lobe. A CT scan of one patient revealed central necrosis in the absence of hemorrhage and cystic change. One patient had calcification and mild enhancement. For the other 6 patients, pre- and post-enhanced CT densities were homogenous and showed marked enhancement (Fig. 1), while the dynamic-contrast enhancement curves of seven lesions (87.5 %) appeared as type III. All lesions showed enhanced capsules in the delayed phase of post-enhancement CT while one lesion appeared plateau-like type. Seven out of eight patients with G1 PHNETs underwent a MRI examination in addition to a CT scan (Table 2). The lesions showed hypointensity on pre-enhanced T1-weighted and homogeneous, mildly high intensity or high intensity on T2-weighted imaging. Enhancements on post-enhanced MRI scans had markedly high (6 cases) and mildly high intensities (1 case). The ADC values in the liver tumors were lower than the surrounding normal liver tissue (1.39 ± 0.20 × 10^−3^ mm^2^/s versus (2.0 ± 0.38 × 10^−3^ mm^2^/s). All lesions showed enhanced capsules in the delayed phase of post-enhancement MRI scans. These data further confirm that G1 lesions were benign or low-grade malignant tumors and distinguishable from hepato-cellular carcinoma.

**Table 1:** CT appearance of PHNET

Pathological grade	Case	Lesion		Location		Unenhanced CT				Contrast-enhanced series (attenuation)						Type of dynamic enhanced curves^b^	
		Single	Multiple	Right	Left	Hypo	Hyper	Homogeneity		Arterial phase^a^		Portal phase^a^		Delayed phase^a^		Type II	Type III
								Yes	No	Markedly hyper	Moderately hyper	Hyper	Iso	Hyper	Iso		
G1	8	8	0	7	1	7	1	6	2	7	1	8	0	8	0	1	7
G2	10	7	3	6	1	10	0	4	6	8	2	8	2	9	1	1	9
G3	11	4	7	2	2	10	1	0	11	9	2	11	0	9	2	1	11

^a^If the tumor showed a cystic or predominantly cystic appearance, the attenuation of contrast-enhanced series on arterial phase, portal phase and delayed phase were measured in the margin region. The type of dynamic enhanced curves were obtained from the margin or solid content

^b^Type I dynamic enhancement curves show lesions with gradual enhancements and peak enhancements in the delayed phase. Type II dynamic enhancement curves show lesions with peak enhancements at the arterial phase and no decrease at the portal phase or delayed phase. Type III curves shows lesions with peak enhancements at the arterial phase and a slight decrease at the portal phase and the delayed phase

**Table 2:** MR appearances of PHNET

Pathological grade	Cases	Lesion		Location		Unenhanced MRI			Post-enhanced MRI				ADC value
		Single	Multiple	Right lobe	Left lobe	T1WI	T2WI		Enhancement		Type of dynamic curves^a^		
						Hypointensse	Mildly hyper-intensity	Markedly hyper-intense	Marked	Mild	Type II	Type III	
G1	7	7	0	6	1	7	5	1	6	1	1	6	1.39 ± 0.20
G2	8	6	2	5	1	8	7	1	8	0	0	8	1.26 ± 0.23
G3	9	4	5	2	2	9	3	6	9	0	0	9	1.14 ± 0.17

^a^Type I dynamic enhancement curves show lesions with gradual enhancements and peak enhancements in the delayed phase. Type II dynamic enhancement curves show lesions with peak enhancements at the arterial phase and no decrease at the portal phase or delayed phase. Type III curves shows lesions with peak enhancements at the arterial phase and a slight decrease at the portal phase and the delayed phase

**Fig. 2:**
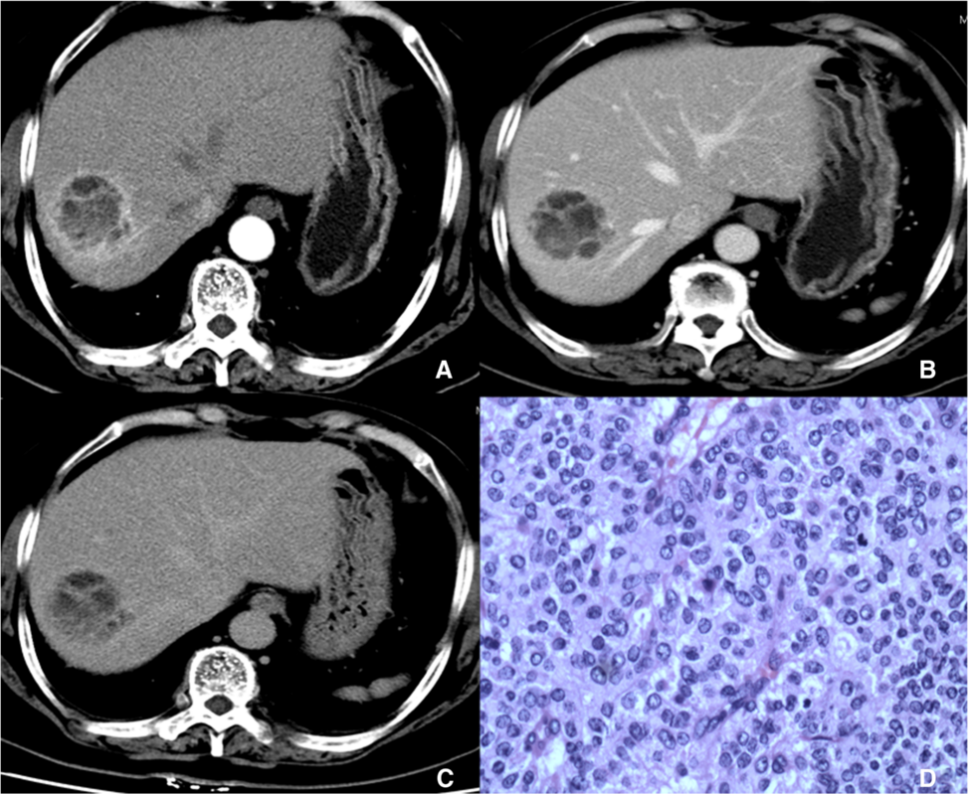
CT scan and histology of grade PHNET. Post-contrast CT image of the arterial phase (**a**), portal venous phase (**b**), and delayed phase (**c**). The PHNET (5.4 × 5.7 cm in size) has marginal ring-like and septal enhancements (right lobe). Arterial phases reveal a wash-in pattern at the periphery of the tumor. Portal phase and delayed phase reveal slight peripheral hyper-attenuation compared with the surrounding liver parenchyma. HE staining shows liver tumor cells arranged in an island. Atypia and nucleoli were not readily apparent but nuclear chromatins were distributed in fine granules (**d**). Mitosis rate was 2–3/10 HPF and blood sinus was abundant in the intestinal with no hemorrhage

For the ten patients with G2 PHNETs, CT scans revealed both single and multiple lesions with necrosis (Table 1). Nodular or marginal-ring enhancements were presented on post-enhanced CT images (Fig. 2). Seven out of ten patients had single lesions that appeared with capsule-enhancement in the delayed phase. One case appeared as cystic and solid and was presumed to be echinococcosis on unenhanced, post-enhanced CT imaging and a pre-operational ultrasound because the patient lived in an area with an echinococcosis epidemic. This case was later confirmed to be a G2 PHNET based on pathology following surgical resection. Three cases had multiple lesions and were misdiagnosed as metastases on CT and MRI scans, but were later determined to be G2 PHNETs based on needle biopsies. Furthermore, MRI revealed necrosis and cystic change in these lesions. Signal intensities were heterogeneous on T1- and T2-weighted imaging with significant necrosis (Fig. 2) and appeared as hypointensity on T1WI and mildly high intentsity on T2WI. The ADC values on diffusion-weighted imaging (DWI) were lower relative to normal liver tissue (1.26 ± 0.23 × 10^−3^ mm^2^/s versus 2.02 ± 0.26 × 10^−3^ mm^2^/s, respectively). Nodular or marginal-ring enhancements were presented on post-enhanced MR images. Dynamic enhanced curves (9/10) with CT and MRI appeared as type III in the tumor margin or solid area.

**Fig. 3:**
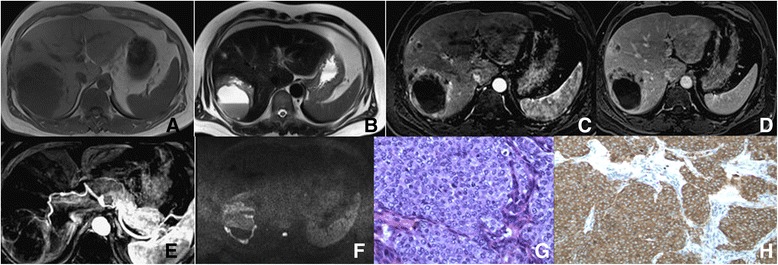
MRI and histology of grade 3 PHNET. Transverse T1-weighted (**a**), transverse T2-weighted (**b**), contrast-enhanced dynamic T1-weighted at the arterial phase (**c**) and delayed phase (**d**). Figure **e** and **f** illustrate liver arterial reconstruction, and transverse diffusion-weighted imaging(b = 800 s/mm^2^, respectively). This grade 3 tumor (8.4 cm in size) was located in the right lobe with a cystic change in the liver wall. Multiple satellite nodules show marked peripheral enhancement in the arterial phase and slight hyper-intensity relative to the surrounding liver parenchyma in the delayed phase. Diffusion-weighted imaging (Figure **f**) shows high peripheral signal intensity, which reflects the diffuse restriction of water. Figure **e** confirms that the tumor blood supply comes from the liver artery. HE staining of liver tumor cells with atypia, varying sizes, multiple mitosis rates, and irregular nucleoli (**g**). Staining for the neuroendocrine marker, synaptophysin (Syn) was positive (**h**). Magnifications: G and H = 100X

Four out of eleven cases with G3 PHNETs were singular and the remaining seven cases appeared as diffuse, multi-nodular or marginal-ring enhancement on CT. Three multi-nodular cases were misdiagnosed as metastases and two cases were misdiagnosed as hepatocellular carcinoma with diffuse intra-liver metastases. This misdiagnosis further suggests that a combination of a detailed clinical history and lab tests are essential in the accurate diagnosis of PHNET. The dynamic-contrast enhancement curves illustrated type III. Hemorrhage was seen in one tumor (Fig. 3) with no evidence of lymph node metastases. However, one case had tumor thrombus in the portal vein in and 10 cases had necrosis, heterogeneous hypo-intensity on T1WI and high intensity or mildly high intensity on T2WI as illustrated by MRI. On T2WI, one case showed the liquid-liquid level and another showed hypo-intensity in the lower part. All patients (9/9) showed multi-nodular or marginal-ring markedly high enhancements on post-enhanced MRI and at the margins or solid area of the tumor dynamic-contrast enhancement curves (type III). ADC values were lower in liver lesions compared to the surrounding normal liver tissue (1.14 ± 0.17 × 10^−3^ mm^2^/s versus 1.97 ± 0.30 × 10^−3^ mm^2^/s, respectively). In one case, tumor thrombus appeared as a filling-defect in the portal vein.

## Discussion

In this work, we found that careful imaging with CT and MRI scans could accurately reflect the histological features of primary hepatic neuroendocrine tumors (PHNETs) and increase the likelihood of a correct diagnosis. A tumor that originates from neuroendocrine cells is a neuroendocrine tumor (NET). There are two types of NETs: neural neuroendocrine tumors and epithelial neuroendocrine tumors. Primary hepatic neuroendocrine tumors (PHNETs) develop in the intrahepatic bile duct epithelium and have an extremely low incidence of 0.17 % [8].

G1 and G2 NETs are low grade neoplasms and have a good prognosis. Neuroendocrine carcinomas (NECs) are G3 neoplasms that are structurally similar to NETs. The neuroendocrine markers synaptophysin (Syn) and chromogranin A (CgA) are diffusely expressed and accompanied by nuclear atypia, focal necrosis, and a high mitosis rate (>20/10 HPF).

Hepatic NECs have a better prognosis than hepatocellular carcinoma [9], but characteristic local invasion, metastases, and serious complications of carcinoid syndrome contribute to the poor outcome of patients with hepatocellular carcinoma [10–12]. Furthermore, positive staining for chromaffin granule (CgA), synaptophysin (Syn), and neuron specific enolase (NSE) are important in the pathological diagnosis of PHNET.

Zhu Z et al. [13] reported that PHNETs appear as a single tumor. Our MRI and CT scans revealed that G1 PHNETs usually have single lesions in the right lobe whereas G3 PHNETs commonly have multiple diffuse lesions or one large tumor accompanied by several satellite lesions. PHNETs are difficult to distinguish from liver cancer [14]. Kim JE et al. [6] reported variation in the CT images of hepatic neuroendocrine tumors that did not correlate with their pathologic diagnoses. In these cases, PHNETs showed arterial enhancements that resembled hepatocellular carcinoma and delayed phase enhancements that resembled a cholangiocarcinoma. In our study, we had six cases of PHNETs (three cases were G2 and three cases were G3) that were misdiagnosed as metastases. After analyzing the rationale behind the misdiagnosis, we found that PHNETS are markedly enhanced on the arterial phase and portal phase, which commonly shows a major lesion surrounded by several satellite nodules. Furthermore, the major lesion usually has discontinuous and irregular capsule-enhancements.

ADC values showed that the PHNET had restricted diffusion compared with the surrounding normal liver, which confirmed that the PHNET was malignant. As PHNET lesions grew in size, the number of intrahepatic lesions increased from single to multiple, focal hemorrhage, necrosis, and portal venous thrombosis became prevalent [15]. Su M et al., [16] analyzed six cases of PHNET with multiple lesions. These tumors appeared as nodular or ring-like enhancements that suggest that PHNET is not of a multifocal origin, which is common to liver cancer.

A rich blood supply from the hepatic artery is one characteristic of PHNETs, which is reflected in the type of dynamic enhancement curves. In our study all lesions were markedly enhanced in the arterial phase and the reconstruction of the arterial phase confirmed a rich blood-supply. However, it is difficult to distinguish PHNETs from other diseases such as primary hepatocellular carcinoma (HCC), hepatic adenoma and focal nodular hyperplasia (FNH), hepatic epithelial angiomyolipoma and liver metastases, which also receive their blood supply the liver artery. Primary hepatocellular carcinoma (HCC) has the typical manifestations of marked arterial enhancement and shows a washout pattern that makes it easy to misdiagnose PHNET with tumor thrombus as HCC. The majority of HCC patients has a history of hepatitis, liver cirrhosis, and increased serum levels of alpha-feto protein (AFP). In contrast, PHNETs show a washout in portal venous phase and higher enhancements in delayed phases compared to the surrounding liver parenchyma. Hepatitis, liver cirrhosis, portal vein thrombosis, and increased serum AFP levels are uncommon in PHNET patients. Hepatic adenoma and focal nodular hyperplasia (FNH) has a portal venous phase enhancement that is equal to or slightly higher than normal liver tissue. Hemorrhage and necrosis are rarely seen in FNH. Individuals who have a history of taking oral contraceptives have a higher incidence of hepatic adenomas [17]. According to Burke C et al., [18] liver-specific contrast agents could be used to distinguish FNH from PHNETs. Chemical shift imaging could be used to differentiate PHNETs from hepatic adenoma based on fat content since adenomas have specific appearances on in-phase and out-of phase T1WI, which have markedly suppressed signal intensities on the out-of phase T1WI FLASH sequence. Liver metastases have a rich blood supply as well, but the characteristic bull’s eye pattern on MRI and CT scans distinguish liver metastases from PHNETs.

One limitation of our study is the small sample size, which results from the low incidence of this rare tumor. Therefore, future studies must include more cases, which will allow for the inclusion of statistical data.

## Conclusion

In conclusion, the appearances of CT and MRI reflect the biologically benign fashion of G1 and G2 PHNETs and the malignant growth of G3 PHNETs. This data could be used to diagnose PHNETS and distinguish grade 1 and 2 tumors from grade 3 tumors, which typically show multiplicity and central necrosis.
